# Raman Spectrum of Follicular Fluid: A Potential Biomarker for Oocyte Developmental Competence in Polycystic Ovary Syndrome

**DOI:** 10.3389/fcell.2021.777224

**Published:** 2021-11-11

**Authors:** Xin Huang, Ling Hong, Yuanyuan Wu, Miaoxin Chen, Pengcheng Kong, Jingling Ruan, Xiaoming Teng, Zhiyun Wei

**Affiliations:** Department of Assisted Reproduction, Shanghai Key Laboratory of Maternal Fetal Medicine, Shanghai First Maternity and Infant Hospital, School of Medicine, Tongji University, Shanghai, China

**Keywords:** polycystic ovary syndrome (PCOS), Raman spectroscopy, metabolic profiles, machine learning, biomarker, embryo development

## Abstract

Polycystic ovary syndrome (PCOS) is a common endocrine and metabolic disorder in reproductive women where abnormal folliculogenesis is considered as a common characteristic. Our aim is to evaluate the potential of follicular fluid (FF) Raman spectra to predict embryo development and pregnancy outcome, so as to prioritize the best promising embryo for implantation, reducing both physiological and economical burdens of PCOS patients. In addition, the altered metabolic profiles will be identified to explore the aetiology and pathobiology of PCOS. In this study, follicular fluid samples obtained from 150 PCOS and 150 non-PCOS women were measured with Raman spectroscopy. Individual Raman spectrum was analyzed to find biologic components contributing to the occurrence of PCOS. More importantly, the Raman spectra of follicular fluid from the 150 PCOS patients were analyzed *via* machine-learning algorithms to evaluate their predictive value for oocyte development potential and clinical pregnancy. Mean-centered Raman spectra and principal component analysis (PCA) showed global differences in the footprints of follicular fluid between PCOS and non-PCOS women. Two Raman zones (993–1,165 cm^−1^ and 1,439–1,678 cm^−1^) were identified for describing the largest variances between the two groups, with the former higher and the latter lower in PCOS FF. The tentative assignments of corresponding Raman bands included phenylalanine and *β* -carotene. Moreover, it was found that FF, in which oocytes would develop into high-quality blastocysts and obtain high clinical pregnancy rate, were detected with lower quantification of the integration at 993–1,165 cm^−1^ and higher quantification of the integration at 1,439–1,678 cm^−1^ in PCOS. In addition, based on Raman spectra of PCOS FF, the machine-learning algorithms via the fully connected artificial neural network (ANN) achieved the overall accuracies of 90 and 74% in correctly assigning oocyte developmental potential and clinical pregnancy, respectively. The study suggests that the PCOS displays unique metabolic profiles in follicular fluid which could be detected by Raman spectroscopy. Specific bands in Raman spectra have the biomarker potential to predict the embryo development and pregnancy outcome for PCOS patients. Importantly, these data may provide some valuable biochemical information and metabolic signatures that will help us to understand the abnormal follicular development in PCOS.

## Introduction

Polycystic ovary syndrome (PCOS) is the most common and complex endocrinopathy, which affects more than 10% of women of reproductive ages (Apridonidze et al., 2005). It is a multi-factorial and heterogeneous syndrome with variable phenotypes, including hyperandrogenism, menstrual irregularity and polycystic ovarian morphology (Rotterdam, 2004). The clinical and biochemical characteristics of PCOS have typical heterogeneity, however, abnormal follicular development to induce anovulation is still the basic important characteristic of PCOS (Fux Otta et al., 2013; Christakou and Diamanti-Kandarakis, 2014). Due to menstrual disorder or anovulation, PCOS patients often have to obtain pregnancy through assisted reproductive technology. However, in clinical practice, sufficient oocytes are usually retrieved from PCOS patients who were under controlled ovarian stimulation (COS) during *in vitro* fertilization (IVF), but high-quality mature oocytes or embryos are limited in number (Wood et al., 2003; Wood et al., 2007). Therefore, understanding the causes of follicle abnormalities and selecting the most promising embryos for transplantation are the prominently urgent tasks to improve the pregnancy rate of PCOS patients.

It is known that metabolomics is an attractive approach to identify and quantify small molecules and could describe both the physiological and pathological states of the organism (Yan et al., 2015). As the important microenvironment for follicular development and oocyte maturation, follicular fluid (FF) is the medium for bi-directional communication between oocyte and the surrounding cells (Matzuk et al., 2002). Accordantly, the metabolomics of follicular fluid will truly reflect the folliculogenesis. In fact, the levels of cytokines, growth factors, proteins, metabolites and non-coding RNAs in FF have been reported to be associated with oocyte quality and pregnancy outcome (O’Gorman et al., 2013; Battaglia et al., 2017; Kollmann et al., 2017). The paucity of high-quality mature oocytes of PCOS patients may due to abnormal endocrine and intra-ovarian paracrine interactions in the follicular fluid microenvironment. Therefore, metabolic profiles of follicular fluid from PCOS patients will not only monitor the smallest biochemical changes in the oocyte development, but also help to provide a specific test for the PCOS diagnosis or embryo selection (Barderas et al., 2011; McRae et al., 2013).

As a noninvasive and label-free method for metabolomics, Raman spectroscopy has started to be increasingly applied in biomedical science. It describes the inelastic scattering of light that provides the unique molecular fingerprints of relevant biological molecules (Li et al., 2012). As it should be, Raman spectroscopy shines a new light on reproductive medicine by investigating complex biochemical interactions or evaluating of living cells and tissue (Mallidis et al., 2014). The first Raman investigations of reproductive organs focused on oncology. In 1992, human cervical, uterine, endometrial and ovarian tissues were examined by Raman technology, and four specific regions of the Raman profile between normal/benign and cancerous states were identified (Liu et al., 1992). There is, nevertheless, a scarcity of studies using Raman spectroscopy to investigate the relationship between metabolic changes and embryo development or its pregnancy outcome during IVF process. In 2007, Seli et al. (2007) firstly investigated the Raman profiles of spent IVF culture media from Day 3 human embryos that implanted or not. Their results confirmed its strong association with the metabolomic profile and clinical outcome. Later, a retrospective study reported that the changes of metabolic footprints in embryo growth medium that could be detected by Raman spectroscopy was related with chromosomal abnormalities in embryos (Liang et al., 2019). Recently, Raman spectroscopy was used in detecting the metabolic changes associated with reproductive related diseases [i.e., endometriosis (Notarstefano et al., 2019), PCOS (Zhang et al., 2021)]. It was found that the vibrational Raman spectroscopy characterization of granulosa cells (GCs) from patients affected by unilateral ovarian endometriosis was abnormal. The altered GCs metabolism and biochemical composition impaired the overall ovarian functions (Notarstefano et al., 2019). For PCOS, the Raman profile of FF was found to be different from that of normal women (Zhang et al., 2021), but its predictive value for embryo development and pregnancy outcome has not been established yet.

In this study, we performed Raman spectroscopy combined with multivariate statistical methods to detect the metabolic changes in follicular fluid from women with PCOS. Based on the specific spectral bands in FF, we also investigated the possibility and accuracy of the Raman biomarkers, and implemented an artificial intelligence (AI) approach to predict the embryo development and clinical outcome for PCOS patient undergoing IVF. Besides the biomarker potential of Raman profiles, the results also suggest the changes of metabolism in FF of PCOS patients. To some extent, it will also reveal the pathogenesis of abnormal folliculogenesis and offer a new potential strategy for PCOS therapy.

## Materials and Methods

The study was approved by the Institutional Ethical Review Board of Tongji University School of Medicine (Reference: TJUSM-2017-0318). Written informed consent was obtained from all patients and the study was approved by the Ethics Review Board of Shanghai First Maternity and Infant Hospital (Reference: 2017-RM-0920).

### Participants and Sample Collection

A total of 300 participants (150 non-PCOS and 150 PCOS) who underwent the conventional *in vitro* fertilization (IVF) in Shanghai First Maternity and Infant Hospital between October 2017 and December 2019 were recruited to obtain informed consent. Eligibility inclusion criteria were: 1) 25–35 years of age; 2) diagnosis of PCOS, according to the revised Rotterdam European Society of Human Reproduction and Embryology/American Society for Reproductive Medicine Criteria (Rotterdam, 2004), where at least two of the followings were fulfilled: chronic oligo-ovulation or anovulation, androgen excess, and polycystic ovaries. The control group had regular menstrual cycles, normal ovary sonographs and normal ovulating; 3) no history of drugs affecting glucose and lipid metabolism, and without any other diseases that affect endocrine hormones, including congenital adrenal hyperplasia, Cushing’s syndrome, endometriosis and androgen-secreting tumors; 4) undergoing the first IVF treatment with GnRH antagonist protocol; 5) males with normal sperm according to the WHO criteria to diminish the chances of failure on embryo development due to defects in the spermatozoa; 6) one blastocyst transferred in fresh cycle or freezing-thawing cycle afterwards, in order to track the pregnancy outcome. Namely, if the oocyte was abnormally fertilized or did not develop into a transferrable blastocyst, its corresponding FF was discarded and the patients was not included in this study. The clinical characteristics of the PCOS and non-PCOS controls are summarized in Table 1.

**TABLE 1:** The clinical characteristics of women with PCOS and non-PCOS control.

	PCOS group (*n* = 150)	Non-PCOS group (*n* = 150)	*p* Value
Age (y)	29.68 ± 3.19	30.64 ± 3.25	0.711
BMI (kg/m^2^)	24.04 ± 3.36	21.54 ± 2.66	**0.034**
Duration of infertility (y)	1.7 ± 1.3	1.6 ± 1.5	0.84
Primary infertility, *n* (%)	38 (59.3%)	41 (56.9%)	0.78
FSH (mIU/ml)	6.64 ± 1.95	6.75 ± 1.21	0.23
LH (mIU/ml)	10.31 ± 8.47	6.62 ± 1.30	**<0.001**
Basal LH/FSH	2.25 ± 0.71	1.21 ± 0.22	**<0.001**
E_2_ (pg/ml)	56.01 ± 13.11	45.59 ± 12.62	0.41
T (ng/ml)	0.72 ± 0.22	0.38 ± 0.13	**<0.001**
*p* (ng/ml)	0.69 ± 0.21	0.54 ± 0.23	0.29
PRL (ng/ml)	14.85 ± 7.77	13.84 ± 6.26	0.118
AFC	18.24 ± 3.25	9.90 ± 2.31	**<0.001**

Note: PCOS, polycystic ovary syndrome; BMI, body mass index; FSH, follicle-stimulating hormone; LH, luteotrophic hormone; E_2_, oestradiol; T, testosterone; *p*, progesterone; PRL, prolactin; AFC, antral follicle count. Data are presented as the mean ± SD. The bold values indicate the significant differences between the two groups (p < 0.05).

All patients who participated in the current study underwent the same long GnRH antagonist stimulation protocol. The details of the stimulation cycle procedure were previously described (Onofriescu et al., 2013). When two or more follicles were at least 18 mm in diameter and the serum E_2_ levels were at least 300 pg/ml per dominant follicle, the patients received 250 μg hCG (Profasi; Serono). Follicular fluid (2–3 ml) was collected from one of the dominant follicles (>18 mm) by vaginal puncture under ultrasound echo-guidance 36 h after hCG administration. Then, the corresponding oocyte isolated from the collected FF was rinsed and inseminated individually to culture and evaluate the development potential. The collected FF samples were centrifuged at 1,000 g for 10 min at 4°C to remove the cells and debris. The supernatant was stored at −80°C for Raman spectra test.

### Embryo Cultivation and Assessment of Blastocyst Quality/IVF Outcome

Approximately 2–3 h after the oocyte retrieval, insemination was performed with Percoll-prepared spermatozoa. One hundred thousand sperms per milliliter were added to each oocyte. The fertilized oocytes were cultured in sequential media of SAGE (CooperSurgical, Leisegang Medical, Berlin) in a single droplet culture (25 μL) covered by mineral oil and cultured at 37°C with 6% CO2 to the blastocyst stage on Day 5–6. The morphological characteristics of the oocytes and embryos were individually recorded. On Day 1 (at 16–18 h after insemination), zygote exhibiting two pronuclei (2 PN) was regarded as normal fertilization and continued to be cultured to the blastocyst stage (Day 5/6), while the zygote exhibiting abnormal fertilization (0 PN or 3 PN) was discarded. And, the blastocysts were scored according to the Gardner scoring system (Gardner et al., 2000). Briefly, blastocysts were evaluated mainly through three indicators: the expansion of the blastocoel cavity, the cohesiveness of the inner cell mass, and trophectodermal cells. First, according to the expansion of the blastocoel cavity, the blastocyst was given a numerical score from 1 to 6. From 1 to 6 points, the expansion of the blastocyst cavity was indicated as follows: 1, the blastocoel was less than half of the volume of the embryo; 2, the blastocoel was more than half of the volume of the embryo; 3, the blastocoel completely filled the embryo; 4, the blastocyst was expanded larger than the early embryo, with a thin zona; 5, a hatching blastocyst with the trophectoderm started to herniate though the zona; 6, a hatched blastocyst which had completely escaped from the zona. Second, the inner cell mass and trophectoderm were assessed for blastocysts with scores at least 3. The scoring criteria for inner cell groups were as follows: A (the highest score), many cells were tightly packed; B, several cells were loosely grouped; C, very few cells existed. Lastly, the trophectoderm was also scored as A (the highest score), B, or C as follows: A, a cohesive epithelium with many cells; B, a loose epithelium with few cells; C, very few large cells. In this study, blastocysts with scores ≥ 3BC (blastocoel cavity score ≥3, and inner cell score ≥ B, and trophectoderm score ≥ C) are regarded as transferrable blastocysts. And, the blastocysts with scores below 3BC were discarded. In order to analyze the relationship between the metabolic components of follicle fluid and the development of the blastocyst, the transferrable blastocysts (score ≥3BC) were divided into two subgroups: high-quality (score ≥4BB) blastocysts and low-quality (score ≥3BC and ≤4BC). The transferrable blastocysts were transferred in fresh cycles or freezing-thawing cycles. Vaginal progesterone was given for 2 weeks after the oocyte retrieval. The presence of an intrauterine gestational sac is defined as clinical pregnancy.

### Raman Spectroscopy

The Renishaw inVia Raman spectrometer (Witec alpha300 R, German) connected to a Zeiss microscope (Axioscope, German) and equipped with a 532 nm laser was used for the spectra collection. The 50x dry lens objective (Zeiss EC Epiplan, German) focused the laser beam with a power of 5 mW. The size of the laser spot was 100 μm. The spectral range was from 50 to 2000 cm^−1^ with a spectral resolution at 1 cm^−1^. The accumulative times of a single point are 8 times (8 s per time). Before each scanning session, silicon wafer was used for calibration (adjusted to 520.5 ± 0.3 cm^−1^ for silicon peak). Briefly, the FF samples from −80°C storage were thawed at room temperature (25°C) for 30 min before analysis. A total of 7 μL of the FF sample was dropped onto a clean place on the surface of the disposable aluminum slide without touching the substrate. For each drop of FF, 5 individual spectra were collected at random spots. The background control spectrum was subtracted from each sample spectrum. The process of Raman analysis took about 10 min per sample.

### Data Processing and Analysis of Raman Spectra

In this study, spectral analysis relied on machine-learning data sets and bioinformatics methods. The full-spectrum pattern recognition was drawn and the characteristic peaks of Raman spectrum which contains abundant material structure information were identified. The raw Raman spectra were preprocessed by subtracting the dark signal background from each spectrum. Spectral normalization was finished by vector normalization of the fingerprint region from 600 to 1,800 cm^−1^ using the Labspec 6 software (Horiba). Mean-centered Raman spectra were obtained by subtracting the means of the spectra from each sample spectrum. Data analysis, statistics and visualization were done in the R environment with the use of in-house scripts. The Raman data was analyzed by the unsupervised PCA method, which could extract key features, reduce the high dimensionality of the data and determine principal components (PCs). The specific wavenumber of Raman shift derived by PCA analysis were corresponding to the molecular bonds or metabolites. Quantification of metabolite concentration was done by integrating individual Raman bands and presented as box plots.

### Machine-Learning Classification

Besides feature extraction to find biological information for PCOS, screening oocyte with high developmental potential and more promising clinical pregnancy rates are often desirable for IVF purposes. In this study, referring to the methods described in the previous report (Wang et al., 2020), the fully connected artificial neural network (ANN) was established to classify oocyte development potential and IVF outcome based on original Raman spectra of follicular fluid.

**FIGURE 1:**
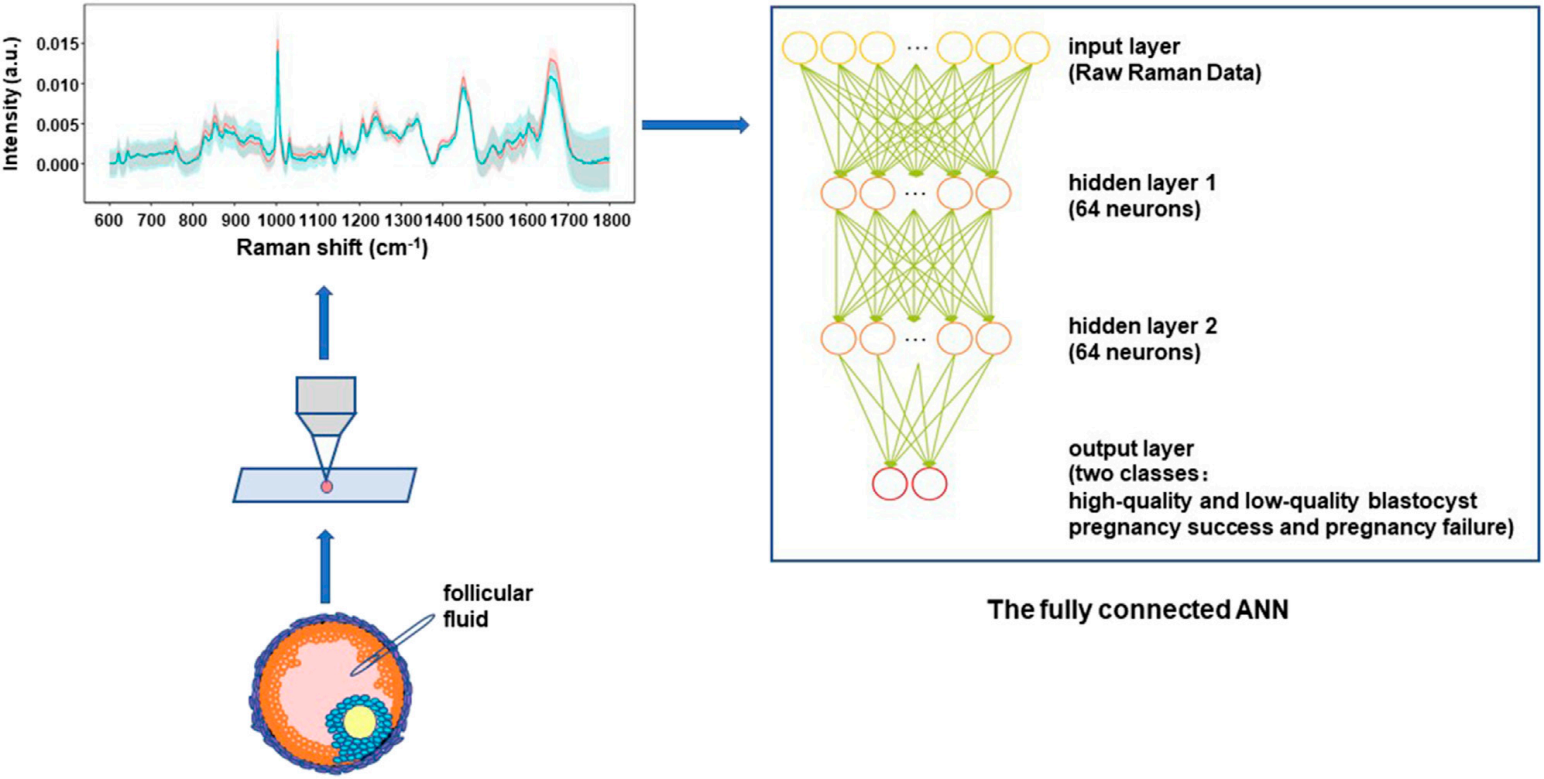
The schematic architecture of the fully connected artificial neural network (ANN) for classification models using Raman spectra. Follicular fluid of PCOS patient were collected and transferred onto an aluminum slide, and tested using Raman spectroscopy. Two full-connection layers were selected for constructing the ANN models in this study. The structure of the ANN included one input layer, 2 hidden layers that contained 64 neurons and 64 neurons, as well as one output layer. The algorithm and principle of the ANN model in details can be found in the Materials and Methods section.

The schematic architecture of the fully connected ANN is illustrated in Figure 1. Because the Raman spectral data is not particularly complex, taking into account the speed and accuracy of the analysis, two full-connection layers were selected for constructing the ANN models in this study. The first layer was the input layer and the input data was one-dimensional intensity data after spectral standardization. The second layer was a fully connected layer of 64 neurons and the third layer was similar as the second layer. An activation function “relu” was associated with each neuron that summed up the outputs from that neuron and transferred them to the next layer in the network, while function “dropout” was used to prevent overfitting. The last layer was the output layer, and the activation function was “softmax.” During the model training process, the loss function was the “crossentropy” and the optimizer was “adam.” The code for building the ANN model was detailed in the Supplementary file. In addition, a very important pre-processing of the raw spectral data was done by using haar wavelets and db4 wavelets to reduce data noise in the process of building the ANN models.

The relationship between input (i.e., Raman spectral pattern of follicular fluid) and output (i.e., the high-quality versus low-quality blastocyst, or pregnancy success versus pregnancy failure) could be learned from the recorded data (training data set) and used to predict the unknown sample (prediction data set). The error threshold was set as 1%. The prediction ability of fully connected ANN analysis was evaluated by “leave-one-out” cross-validation. Briefly, all the data points except one were employed to train the model and a prediction was made for that one at each time. The information of sample ID, replicate number, morphological score and the pregnancy outcome were added as features to train the fully connected ANN models. All models were constructed in a Python 3.3 environment.

For each classification model, the precision, sensitivity, F1 score as well as an overall accuracy rate were calculated. Specifically, precision is the ability of a classifier to not label a negative sample as positive. Sensitivity is the ability of a classifier to find all of the positive samples in one class. F1 score is a weighted mean of the precision and the sensitivity, where 1 is the best value and 0 the worst. And, the performance of the established model was also evaluated by the area under the receiver operating characteristic (ROC) curve (AUC) based on a five-fold cross-validation strategy, which divided the data into five parts, training four parts in turn, and the remaining was used to estimate the performance of the models.

## Results

### Raman Spectral Analysis Unveils Metabolic Differences in Follicular Fluid of PCOS

**FIGURE 2:**
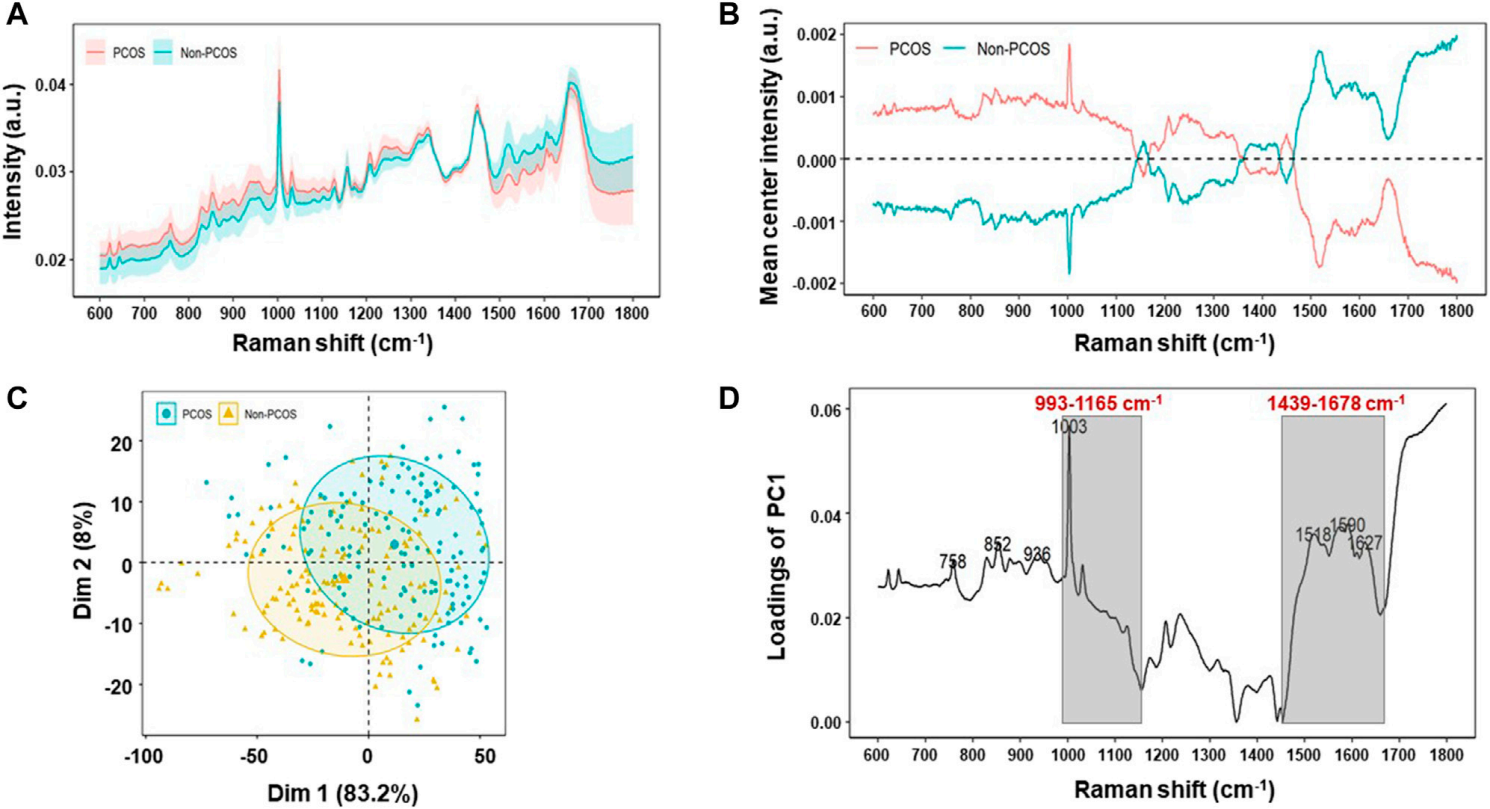
Raman profiling of follicular fluid of PCOS and non-PCOS. **(A)** Averaged Raman spectra of follicular fluid of PCOS (*n* = 150) and non-PCOS (*n* = 150) patients. Shaded areas represent the standard deviations. **(B)** Mean-centered Raman spectra differences between PCOS and non-PCOS groups by subtracting means from all samples. **(C)** Principal component analysis (PCA) of all Raman Spectra to show the clustering of PCOS and non-PCOS groups. **(D)** Raman wavenumber loading plots of contributions along dimension 1 of the PCA, where grey boxes indicate the two Raman zones responsible for the most significant variances between the two groups by comparing the quantified spectral intensity values.

In this study, Raman spectra of 150 PCOS and 150 non-PCOS patients were obtained by analyzing follicular fluid samples. The fingerprint region from 600 cm^−1^ to 1800 cm^−1^ was calculated for analysis, which typically contained the most essential biological information (Li et al., 2012). In accordance with the previous study (Zhang et al., 2021), the raw Raman spectra data of the FF samples from PCOS patients showed no significantly different standard deviation/variation by compared to those of non-PCOS patients (Figure 2A). Then, Raman spectra from all samples were averaged and subtracted from each sample at each wavenumber, and the mean values were assembled for each group to obtain mean-centered spectra. The results show that the mean-centered spectra are significantly different between the two groups (Figure 2B). The corresponding Raman bands and their tentative assignments are summarized in Table 2. Some signatured bands can be assigned to phenylalanine (1,003 cm^−1^), C-C, C-N stretching (protein) (1,156 cm^−1^), β -carotene accumulation (C-C stretch mode) (1,516 cm^−1^), and C=O (1,668 cm^−1^) (Tarcea et al., 2007).

**TABLE 2:** Peak assignments in the Raman spectra from follicular fluid of PCOS patients.

Peak (cm^−1^)	Assignation
758	Tryptophan
	Ethanolamine group
	Phosphatidylethanolamine
852	Proline, hydroxyproline, tyrosine
	Tyrosine ring breathing
	Glycogen
936	C-C stretching mode of proline and valine and protein backbone (*α*-helix conformation)/glycogen (protein assignment)
	*p*(CH3) terminal, proline, valine + ν(CC) *α* -helix keratin (protein assignment)
1,003	Phenylalanine, C-C skeletal
1,156	C-C, C-N stretching (protein)
1,516	β-carotene accumulation (C-C stretch mode)
1,518	v (C=C), porphyrin
	Carotenoid peaks due to C-C and conjugated C=C band stretch
1,590	Carbon particles
1,627	C_α_ = C_α_ stretch
	Amide C=O stretching absorption for the β-form polypeptide films
1,668	Carbonyl stretch (C=O)
	Cholesterol ester

**FIGURE 3:**
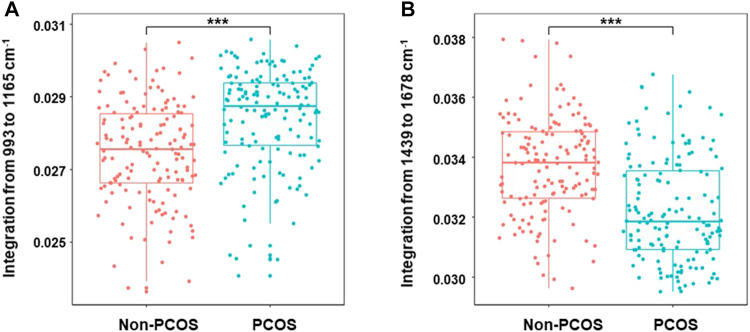
Boxplots of Raman band integration of PCOS and non-PCOS samples. The significant differences at 993–1,165 cm^−1^ **(A)** and 1,439–1,678 cm^−1^ **(B)** were shown between PCOS and non-PCOS groups. The rectangle in the box plots represents the second and the third quartiles, with the line inside representing the median. The lower and upper quartiles are drawn as lines outside the box. Sample means were compared by Welch 2-sample *t* test for unequal variances. ^***^ indicates *p* < 0.001.

All of the spectra were analyzed by unsupervised PCA to reduce the high-dimensional Raman dataset and transform data into appropriate variables that conferred biologic information. A spectral range of 600–1,800 cm^−1^ was used to minimize the effects of uneven baseline. As shown in Figure 2C, PCOS and non-PCOS have two clusters with some overlap. The overlap may be due to the close similarity of follicular fluid between PCOS and non-PCOS patients. In order to find biomarkers which can best separate the two groups and provide diagnostic advice for PCOS, a loading plot, which is along dimension 1, was made to assign each Raman wavenumber to indicate significant Raman bands. Then, by scanning all available zones based on the bands and calculating the significance of each zone on discriminating PCOS vs. non-PCOS, two Raman zones (993–1,165 cm^−1^ and 1,439–1,678 cm^−1^) were identified as the ones with the strongest significance (Figure 2D). The quantification of metabolite concentration between PCOS and non-PCOS groups were also presented at box plots by integrating individual Raman bands at the two Raman zones (993–1,165 cm^−1^ and 1,439–1,678 cm^−1^). The results showed that the quantification of integration at 993–1,165 cm^−1^ was found to be higher in PCOS FF samples (0.0286 ± 0.001) compared with non-PCOS FF samples (0.0274 ± 0.001; *p* < 0.001). And, the quantification of the integration at 1,439–1,678 cm^−1^, on the other hand, showed a higher content in non-PCOS FF samples (0.0337 ± 0.001) than in PCOS FF samples (0.0319 ± 0.001; *p* < 0.001) (Figure 3). All the results suggest that there are real different metabolic patterns in follicular fluid of PCOS patients.

### Special Raman Spectral as Biomarkers for Predicting Oocyte Development and IVF Outcome in PCOS Patients

**FIGURE 4:**
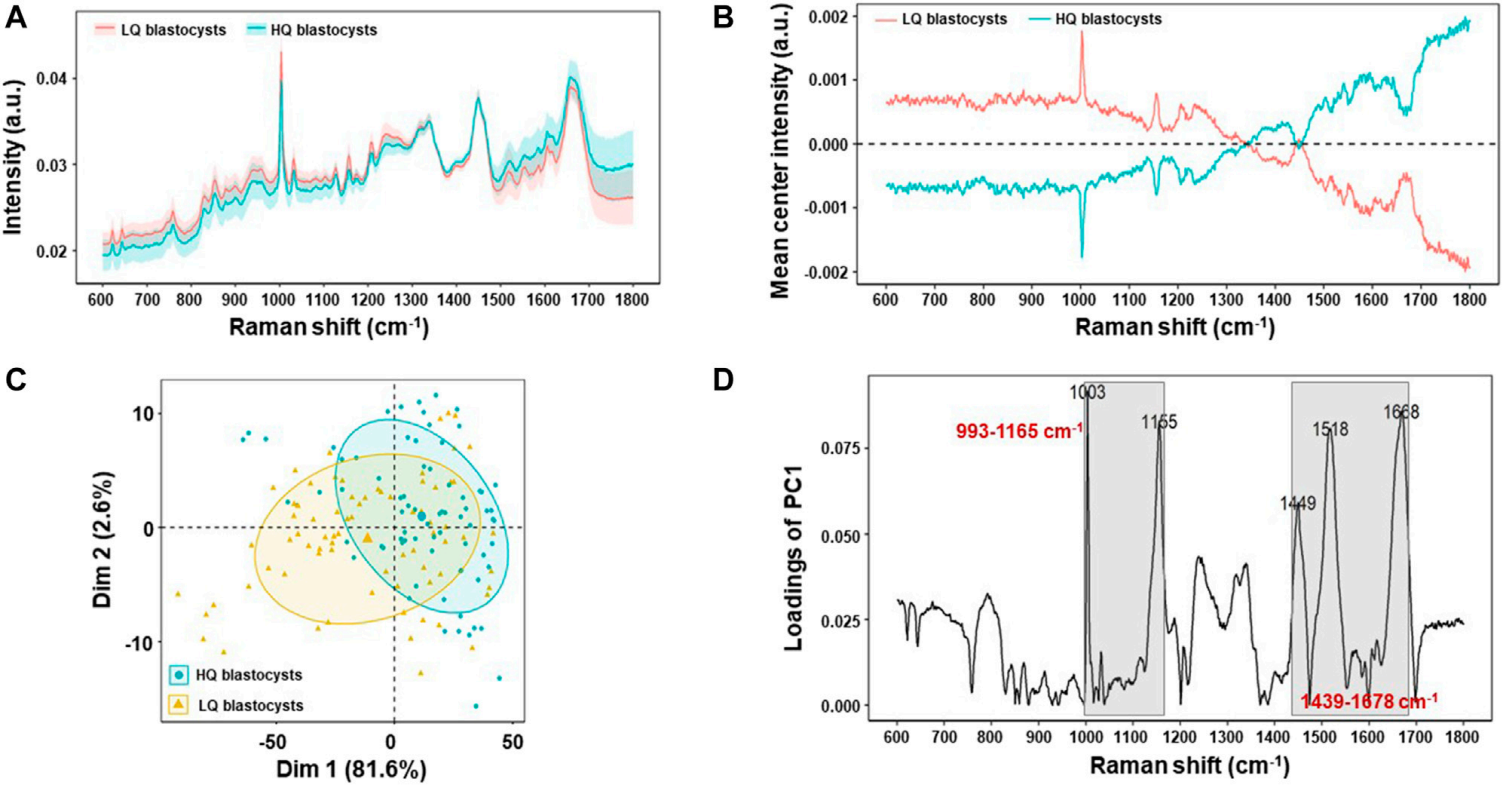
Raman profiling of follicular fluid between high-quality blastocysts group (HQ group) and low-quality blastocysts group (LQ group) of PCOS. **(A)** Averaged Raman spectra of follicular fluid of HQ group (*n* = 75) and LQ group (*n* = 75) patients. Shaded areas represent the standard deviations. **(B)** Mean-centered Raman spectra differences between HQ group and LQ groups by subtracting means from all samples. **(C)** Principal component analysis (PCA) of all Raman Spectra to show the clustering of HQ group and LQ groups. **(D)** Raman wavenumber loading plots of contributions along dimension 1 of the PCA, where grey boxes indicate the two Raman zones responsible for the most significant variances between the two groups by comparing the quantified spectral intensity values.

**FIGURE 5:**
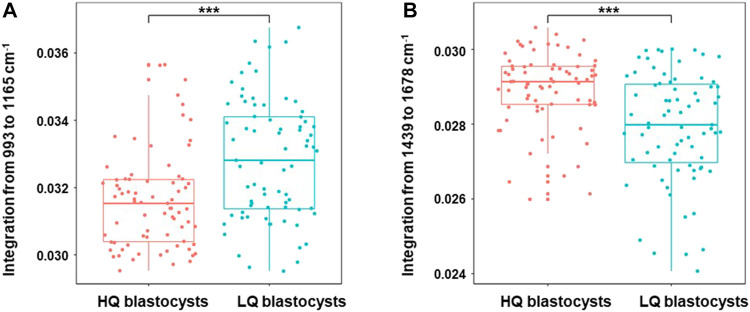
Boxplots of Raman band integration of high-quality blastocysts group and low-quality blastocysts group in PCOS. The significant differences at 993–1,165 cm^−1^ **(A)** and 1,439–1,678 cm^−1^ **(B)** were shown between high-quality blastocysts and low-quality blastocysts groups in PCOS. The rectangle in the box plots represents the second and the third quartiles, with the line inside representing the median. The lower and upper quartiles are drawn as lines outside the box. Sample means were compared by Welch 2-sample *t* test for unequal variances. ^***^ indicates *p* < 0.001.

As the follicular fluid is the direct environment of oocyte, the changes of metabolism in FF will affect oocyte development. In order to investigate whether the Raman spectra were related to the oocyte development, the special Raman spectra were further compared between two subgroups of the transferrable PCOS blastocysts (*n* = 150), according to their morphological scores: 1) high-quality blastocysts group (HQ, ≥ 4BB) (*n* = 75), which included 58 blastocysts formed on Day 5 and 17 ones formed on Day 6, and 2) low-quality blastocysts group (LQ, ≥3BC and ≤4BC) (*n* = 75), which were all formed on Day 6. The raw Raman spectra data and the mean-centered spectra of the FF samples from the two subgroups (HQ group or LQ group) of PCOS were shown in Figure 4 A and B. It indicated that the mean-centered spectra are significantly different between the two subgroups, while the raw Raman spectra data had no significant difference. Unsupervised PCA was also applied to find the significant Raman bands between the two subgroups (Figure 4C) and a loading plot was made along dimension 1 to assign each Raman wavenumber to indicate significant Raman bands. Two Raman zones (993–1,165 cm^−1^ and 1,439–1,678 cm^−1^), same as in the above section (Figure 2D), were also identified as the most significant differences between HQ and LQ subgroups (Figure 4D). As shown in Figure 5, the quantification of the integration at 993–1,165 cm^−1^ was lower in HQ group (0.0316 ± 0.001) compared with LQ group (0.0328 ± 0.001; *p* = 1.07 × 10^–5^). On the other hand, the quantification of the integration at 1,439–1,678 cm^−1^ showed a higher content in HQ group (0.0292 ± 0.001) than LQ group (0.0279 ± 0.001; *p* = 1.92 × 10^–6^).

**FIGURE 6:**
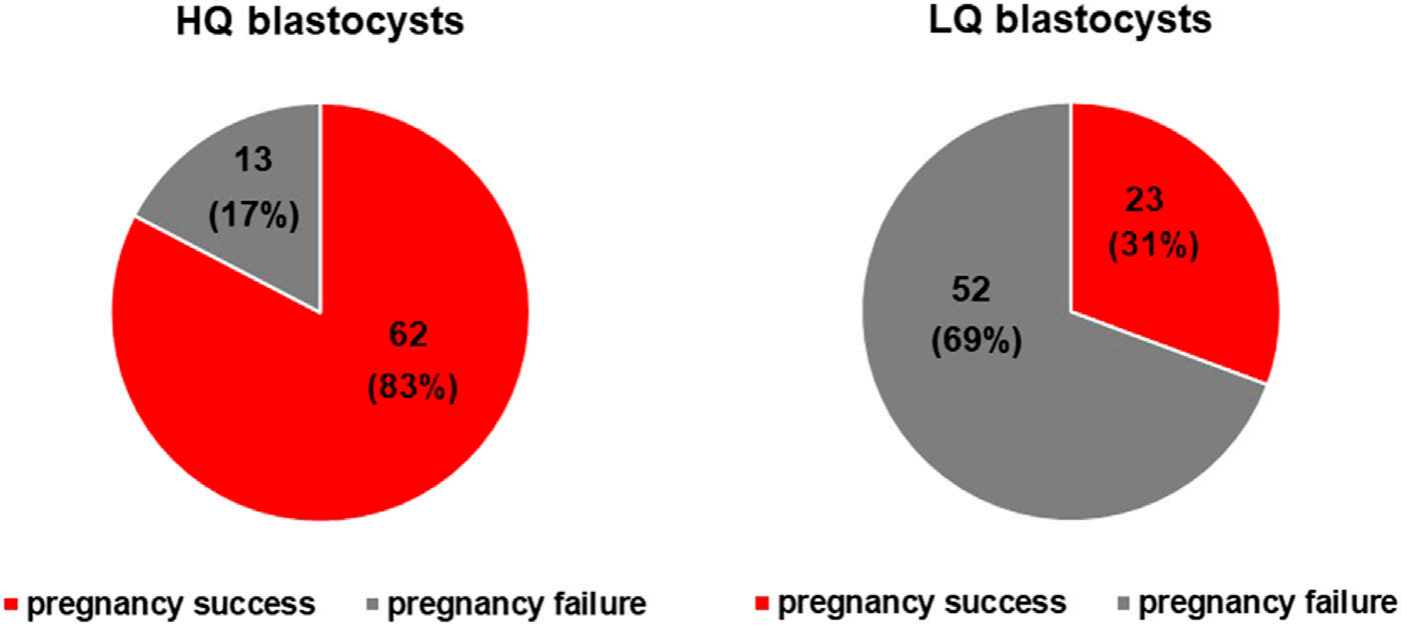
The observation of a connection between blastocyst development and pregnancy outcome. A one-on-one observations of blastocyst development and pregnancy outcomes was made. There are a total of 150 transferrable blastocysts of PCOS patients, including 75 high-quality blastocysts (≥4BB) and 75 low-quality blastocysts (≥3BC and ≤4BC). Of the 75 high-quality blastocysts, 62 successfully conceived, and the clinical pregnancy rate was about 83%. That is, even if a high-quality blastocyst was transplanted, 17% of patients failed to conceive. On the other hand, of the 75 low-quality blastocysts, 23 successfully conceived, and the clinical pregnancy rate was about 31%.

**FIGURE 7:**
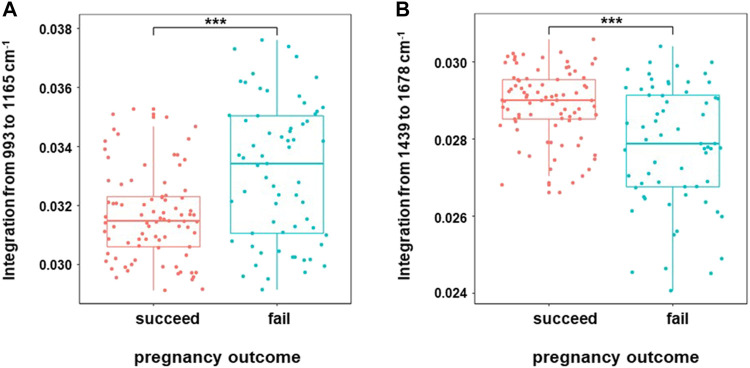
Boxplots of Raman band integration of successful pregnancy group and failed pregnancy group in PCOS. The significant differences at 993–1,165 cm^−1^ **(A)** and 1,439–1,678 cm^−1^ **(B)** were shown between the two groups in PCOS. The rectangle in the box plots represents the second and the third quartiles, with the line inside representing the median. The lower and upper quartiles are drawn as lines outside the box. Sample means were compared by Welch 2-sample *t* test for unequal variances. ^***^ indicates *p* < 0.001.

Embryo quality is a key factor affecting pregnancy outcome. In this study, we tracked the pregnancy outcome of each transferred blastocyst. As shown in Figure 6, there are a total of 150 transferrable blastocysts from PCOS patients, including 75 high-quality blastocysts (≥4BB) and 75 low-quality blastocysts (≥3BC and ≤4BC). Of the 75 high-quality blastocysts, 62 successfully conceived, and the clinical pregnancy rate was about 83%. This indicated that even if a high-quality blastocyst was transplanted, 17% of patients failed to conceive. On the other hand, of the 75 low-quality blastocysts, 23 successfully conceived, and the clinical pregnancy rate was about 31%. In order to investigate the relationship of the special Raman spectra and IVF outcome in PCOS, the 150 Raman spectra of PCOS samples were divided into pregnancy success group (*n* = 85) and pregnancy failure group (*n* = 65). The raw Raman spectra data and the mean-centered spectra of the FF samples from the pregnancy success group and pregnancy failure group are roughly the same as those of high-quality blastocysts group and low-quality blastocysts group (data is not repeated). In order to further determine the Raman spectral difference between the two groups (pregnancy success group and pregnancy failure group), the quantified spectral intensity values were also compared. It indicated that the quantification of Raman spectra (993–1,165 cm^−1^ and 1,439–1,678 cm^−1^) showed the same significantly different between pregnancy success and pregnancy failure groups (*p* < 0.001) (Figure 7). In line with the trend of the same two Raman zones correlating with blastocyst development, the quantification of the integration at 993–1,165 cm^−1^ was lower in pregnancy success group (0.0316 ± 0.001) compared with pregnancy failure group (0.0335 ± 0.002; *p* = 1.36 × 10^–5^), and the quantification of the integration at 1,439–1,678 cm^−1^ was higher in pregnancy success group (0.0289 ± 0.001) than that in pregnancy failure group (0.0278 ± 0.001; *p* = 1.98 × 10^–6^).

### Machine-Learning Models Based on Raman Spectra Classify Blastocysts Development and Pregnancy Outcome With High Performance in PCOS

In this study, based on the 150 Raman spectra of PCOS follicular fluid samples, the two fully connected ANN classification models were computed to predict the blastocyst development and clinical pregnancy, respectively. The spectra of different subgroups (HQ or LQ blastocyst; pregnancy success or pregnancy failure) were randomly split into training set and the testing set in a ratio of 4:1. The training set was used to train a classification model and the testing set was used to evaluate the model performance. Specifically, for the classification model to predict blastocyst development, Raman spectra of 150 PCOS samples were divided into high-quality blastocysts (HQ) (*n* = 75) and low-quality blastocysts (LQ) (*n* = 75) groups. Of them, Raman spectra of 100 samples (50 high-quality blastocysts and 50 low-quality blastocysts) were used to train a classification model and the remaining 50 spectra (25 high-quality blastocysts and 25 low-quality blastocysts) were input as the testing set to evaluate the model performance. At the same time, to construct the classification model to predict the IVF outcome, the same Raman spectra of 150 PCOS samples were divided into pregnancy success (*n* = 85) and pregnancy failure (*n* = 65) groups. Of them, 100 spectra (50 pregnancy success and 50 pregnancy failure) were used to train a classification model and the remaining 50 spectra (35 pregnancy success and 15 pregnancy failure) were input as the testing set to evaluate the model performance.

**FIGURE 8:**
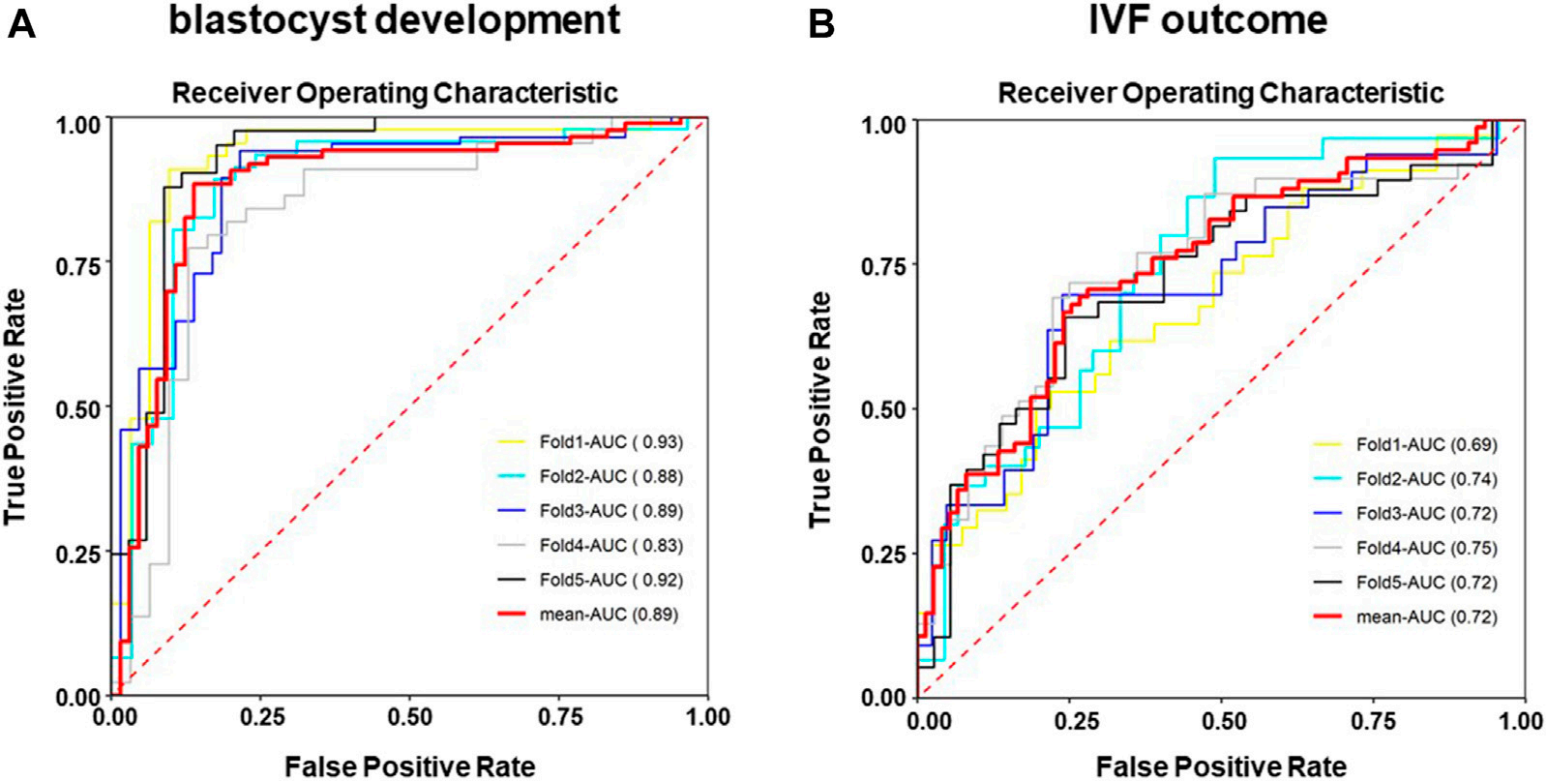
Receiver operating characteristic (ROC) curves of the ANN classification models after randomizing the sample data set. The performance of the established model was evaluated by the area under the receiver operating characteristic (ROC) curve (AUC) based on a five-fold cross-validation strategy, which divided the data into five parts, training four parts in turn, and the remaining was used to estimate the performance of the models. **(A)** ROC analysis of the ANN model for classifying the blastocyst developmental potential. **(B)** ROC analysis of the ANN model for classifying the IVF outcome.

The results of ANN models were presented in Table 3. As shown in Table 3A, 23 out of 25 high-quality blastocysts (F1 score 0.9020) and 22 out of 25 low-quality blastocysts (F1 score 0.8980) were assigned correctly. For classifying clinical pregnancy outcome (Table 3B), the ANN model was able to correctly assign 25 out of 35 pregnancy success spectra and 12 out 15 pregnancy failure spectra, with F1 scores of 0.7937 and 0.6486, respectively. Noticeably, the accuracy of the ANN model for predicting the blastocysts development is 90.00%, while 74.00% for predicting the IVF outcome. Furthermore, ROC analysis resulted in AUCs of 0.89 ± 0.039, sensitivity of 0.692 ± 0.037 and specificity of 0.948 ± 0.031 for the blastocyst developmental ANN model (Figure 8A) and ROC analysis resulted in AUCs of 0.72 ± 0.023, sensitivity of 0.738 ± 0.049 and specificity of 0.63 ± 0.043 for the ANN model predicting the IVF outcome (Figure 8B).

**TABLE 3:** Confusion matrix and performance evaluation of ANN classification models for an independent testing set of 50 Raman spectra.

A						
	Confusion matrix		Performance evaluation			
Sample count	Predicted HQ- blastocysts	Predicted LQ- blastocysts	Precision	Sensitivity	F1 score	Accuracy
Actual HQ- blastocysts	23	2	88.46%	92.00%	0.9020	90.00%
Actual LQ- blastocysts	3	22	91.67%	88.00%	0.8980	
B						
Sample count	Predicted pregnancy success	Predicted pregnancy failure	Precision	Sensitivity	F1 score	Accuracy
Actual pregnancy success	25	10	89.29%	71.43%	0.7937	74.00%
Actual pregnancy failure	3	12	54.55%	80.00%	0.6486	

Note: Models were trained from a training set of 100 Raman spectra.

ANN, is artificial neural network.

## Discussion

As a complex endocrine disease, the application of metabolomics gives a promising insight into the research on PCOS. There has been a growing number of studies on abnormal metabolites in follicular fluid in PCOS patients (Zhao et al., 2015; Zhang et al., 2017; Liu et al., 2019; Sun et al., 2019; Chen et al., 2020; Xu et al., 2020; Hou et al., 2021). Nevertheless, almost all of the studies on PCOS metabolomics aimed to identify metabolic alterations between PCOS and healthy controls, expecting to provide novel ways for the diagnosis and treatment of PCOS. There has been little emphasizing the clinical value of abnormal metabolites in PCOS FF on oocyte development and pregnancy outcome.

To our best knowledge, this is the first report to explore the metabolomics profiles with the use of Raman spectroscopy to perform the machine-learning models for embryo viability selection in PCOS patients. Currently in clinical practice, visual morphology assessment is routinely used for evaluating of embryo quality and selecting the “considerably good” blastocyst for transfer. However, such subjective assessment has nonnegligible inconsistency among embryologists, and as a result, the success rate remains unsatisfactory. Moreover, in the situation where multiple blastocysts have the same morphological scores, the “blind selection” will increase the chances of pregnancy failure and repeated transplants, heavying the financial and psychological burden on patients. Therefore, a more objective and accurate prediction method or model is needed for embryo selection.

Machine-learning approaches are increasingly applied to improve prediction models for clinical decision making (Deo, 2015), and are also thought to have a significant advantage to predict embryo quality reliably (Blank et al., 2019). In this study, we implemented an AI approach based on the fully connected artificial neural network (ANN), which is a state-of-the-art machine-learning architecture with higher computational efficiency, stronger fault tolerance, and better resistance to over-fitting (LeCun et al., 2015). The effectiveness and success of the machine-learning is attributed to several factors, including the sensitivity of the Raman system, the quality of the spectra, the choice of the machine-learning algorithm and the sufficient training datasets. Hereon, the accuracy of the ANN model for predicting the blastocysts development potential is as high as 90%, and the AUC is also up to 0.88. Although our research is still somewhat limited by moderate sample size (FF is more often pooled from multiple follicles of the same patient in clinical practices, and thus *N* = 300 individual FF is so far the largest sample size in literatures), the results indicated that the machine-learning of ANN model is highly effective and promising. It is conceivable that in the future this approach will be more sensitive and robust with higher quality of Raman spectroscopy, ever-improved and advanced algorithms, and increased training datasets. As expected, the accuracy for predicting IVF outcome is less well-performed, as more factors beyond follicles, including genomic stability of the embryo and the endometrial receptivity, are also involved to affect the pregnancy outcome.

The outstanding feature of metabolomics is that, unlike genomics and proteomics, it indicates not only a genetically determined phenotype, but also the differences induced by other factors (i.e., age, diet, or physical activity) (Lindon et al., 2004; Diamanti-Kandarakis et al., 2006; Luque-Ramirez et al., 2006; Atiomo et al., 2009). The metabolic profiles of follicle fluid from PCOS patients reflect both the pathological and physiological states of the abnormal follicle development. Consistent with previous reports (Lane and Gardner, 2005; Gardner et al., 2011), our findings proved that high-quality blastocysts development depends on nutrients in follicular fluid and a complex metabolic activity is involved. The proper metabolic activity will further affect pregnancy outcomes. It is worthy to mention that two prominent spectra (1,003 cm^−1^ and 1,516 cm^−1^) located in the two clinically relevant Raman zones (993–1,165 cm^−1^ and 1,439–1,678 cm^−1^) are assigned to phenylalanine and *β* -carotene, respectively (Tarcea et al., 2007). Agreeing with the previous opinion (Li et al., 2018), we suggest that the two prominent spectra (1,003 cm^−1^ and 1,516 cm^−1^) are related with the metabolic activity of embryos and considered as biomarkers for embryonic development.

Most meaningfully, the metabolomic analysis of follicle fluid not only helps us to find potential biomarkers to select the high-quality embryos and predict the IVF outcome, but also, to some extent, reveals the abnormal mechanism of follicle development of PCOS. For instance, phenylalanine is one of the amino acids that inhibit hamster 1-cell embryo development *in vitro* and is harmful for blastocyst formation in pigs (McKiernan et al., 1995; Booth et al., 2007). Moreover, the concentrations of phenylalanine in embryo culture media are used as biomarkers for clinical pregnancy in humans (Zhao et al., 2013). Our results also indicate that the greater vibration at 1,004 cm^−1^ of phenylalanine in FF is a warning sign for oocyte development in PCOS, and are in line with the previous views that blastocyst development is modulated by amino acids, and the ideal environment for embryonic development needs the supplement of suitable amino acids (Gardner, 1998). It’s worth mentioning that abnormal amino acids are associated with the occurrence of chromosome aneuploidy during human preimplantation embryo development *in vitro* (Picton et al., 2010). It requires further study of whether the elevated phenylalanine in PCOS FF causes chromosomal abnormalities in oocytes, which affects blastocyst development.

Another substance, *β* -carotene, is noticeably greater in follicular fluid for the oocytes with high developmental potential in PCOS patients. *β* -carotene, a precursor to vitamin A, has been postulated to contribute to follicular growth. A positive correlation has been observed between the plasma vitamin and *β* -carotene concentrations and the number of transferable embryos *in vitro* fertilization process (Sekizawa et al., 2012). And, it has been proposed that β -carotene acts as an antioxidant in lipid phases by quenching singlet oxygen and scavenging the peroxyl radical (Ikeda et al., 2005). As known, chronic low-grade inflammation along with increased oxidative stress has been suggested as a key contributor of the pathogenesis and development of PCOS (Artimani et al., 2018). Inappropriate microenvironment conditions can decrease the oxidative metabolism and further underpins the complexity in linking embryo metabolism and viability (Krisher et al., 2015). Based on the fact that high-quality blastocyst needs more *β* -carotene, we propose that enough extracellular *β* -carotene in the follicular fluid could protect oocytes from reactive oxygen species-mediated cytotoxity, thereby enhancing the developmental competence of oocytes. Besides antioxidation, *β* -carotene also has other unique roles in reproduction. The corpus luteum is a steroidogenic tissue that is highly rich in *β* -carotene, and *β* -carotene plays a very important role in regulating the luteal function (Arikan and Rodway, 2000). The levels of *β* -carotene in follicular fluid has been reported to positively correlated with plasma progesterone level as well (Haliloglu et al., 2002). Similar positive relationship also exists in the corpus luteum (Talavera and Chew, 1988). Therefore, we also propose that lower β -carotene levels of follicular fluid might be the main reason for luteal insufficiency and difficult pregnancy in PCOS patients.

## Conclusion

To clarify the effect of abnormal follicular microenvironment on oocyte development in PCOS patients, we developed a noninvasive and label-free rapid strategy (about 10 min per sample) to use Raman spectroscopy to detect metabolic footprint of follicular fluid. Analysis based on Raman spectra showed significant differences in metabolic profiles of follicular fluid between PCOS and non-PCOS patients. Two Raman zones (993–1,165 cm^−1^ and 1,439–1,678 cm^−1^) were identified for describing the largest variances between the two groups. The tentative assignments of corresponding Raman bands 1,003 cm^−1^ (phenylalanine) and 1,516 cm^−1^ (*β* -carotene accumulation) were proposed to be highly promising biomarkers to evaluate blastocysts viability and predict the IVF outcome. The fully connected ANN models successfully classified blastocysts development potential with high accuracy. In summary, our data suggest that noninvasive method of Raman spectroscopy might be a useful tool to identify and predict the embryo developmental potential, and more importantly, to prioritize the best promising oocyte for IVF, reducing both physiological and economical burdens of PCOS patients by avoiding unnecessary implanting cycles.

## Data Availability

The original contributions presented in the study are included in the article/Supplementary Material, further inquiries can be directed to the corresponding authors.
